# Efficient DNA extraction from cytogenetic suspensions: A new possibility for obtaining DNA, with potential applications in studies of molecular markers

**DOI:** 10.1371/journal.pone.0335898

**Published:** 2025-11-07

**Authors:** Geórgia Liz Monteiro Sant’ana-Arruda, Paulo Cesar Venere, Daniela Cristina Ferreira

**Affiliations:** 1 Graduate Program in Ecology and Conservation of Biodiversity, Universidade Federal de Mato Grosso, campus Universitário de Cuiabá, Avenida Fernando Correia da Costa, Cuiabá, Mato Grosso, Brazil; 2 Departamento de Biologia e Zoologia, Universidade Federal de Mato Grosso, campus Universitário de Cuiabá, Avenida Fernando Correia da Costa, Cuiabá, Mato Grosso, Brazil; DePaul University, UNITED STATES OF AMERICA

## Abstract

The present study demonstrates the viability of extracting DNA for genomic analyses from cell suspensions prepared for cytogenetic analyses, specifically, samples fixed in Carnoy’s solution (3:1 methanol: acetic acid) and stored at –20 °C for more than 10 years. Cell suspensions from 27 specimens of fish of the subfamily Loricariinae, which had been prepared originally for cytogenetic studies, were used to test a DNA extraction procedure based on a routinely-used protocol with specific minor adjustments. The spectrophotometric and electrophoretic analyses of the extracted samples revealed DNA at good concentrations (5.9–1739 ng.μL^-1^) and high purity (A260/A280 ratios of 1.75–2.07), indicating negligible contamination and no significant fragmentation. The integrity of the material was confirmed by the successful amplification by PCR of four different genes (COI, 5S, 18S, and RAG2), with the COI gene being sequenced efficiently. These results demonstrate that DNA can be extracted from samples collected for other purposes, even after long-term storage, producing high-quality genomic DNA from sources that might otherwise be overlooked. The exploitation of these samples as a source of DNA represents a useful potential strategy for the acquisition of material for analysis when fresh samples are scarce or difficult to obtain. This novel approach expands considerably the possibilities for retrospective molecular studies, especially in the fields of conservation and systematics.

## Introduction

The development of fast, efficient, and low-cost protocols for the extraction of DNA from different types of materials is fundamental to the advancement of molecular biology. Various cytological preparations originally intended for cytogenetic analyses—even those stored for extended periods—may represent an important source of DNA for genomic studies. In this context, the quality and the quantity of the DNA extracted from these sources can both have a direct influence on the eventual success of downstream applications, such as the *in vitro* amplification of specific genomic regions via Polymerase Chain Reaction (PCR) and enzymatic digestion [1,2]. A number of scientific fields depend on the efficient extraction of DNA for analysis, including molecular systematics, the identification of species, forensic medicine, criminal investigations, paternity testing, and the detection of tumor markers or infectious agents [3,4].

Most of the available protocols for the extraction of DNA, such as those described by Jobes et al. [5], Aljanabi and Martinez [1], and Cheng et al. [6], require tissue samples that have been stored in good conditions, and generally involve time-consuming processing procedures that have a high risk of degrading the DNA. The available commercial DNA extraction kits are also often either expensive or relatively difficult to obtain in developing countries [2]. In this context, the development of a simple, rapid, and low-cost protocol for the extraction of different types of DNA is not only essential for molecular studies, but is also highly desirable when large numbers of samples need to be processed [2–4].

Obtaining biological material for phylogenetic, systematic, or phylogeographic studies can be complex and costly, often leading to the exclusion of key species due to the lack of DNA samples [7]. These difficulties can be exacerbated by factors such as the intrinsic rarity of some species, the challenges of collecting specimens in remote and isolated areas, the need for specialized taxonomic knowledge for the accurate identification of specimens, and even the fact that some species are already extinct [8]. These challenges may be further accentuated by ongoing habitat degradation and the resulting loss of biodiversity [7].

Given these considerations, the present study describes a protocol for the extraction of DNA from samples obtained from fish for cytogenetic preparations, for which they were fixed in Carnoy’s solution (methanol and acetic acid at a ratio of 3:1) and stored at −20°C for more than 10 years. It is important to note here that the potential for the extraction of DNA from samples of cells stored for more than a decade paves the way to a vast reserve of material collected for a range of other, unrelated purposes. The extraction protocol described here expands significantly the diversity of potential sources for the extraction of DNA for molecular studies that would otherwise lack an adequate quantity of samples for analysis.

## Materials and methods

### Acquisition of the samples

The present study was based on the processing of a total of 27 cytogenetic suspensions extracted from fish specimens of the subfamily Loricariinae. These samples were initially collected for conventional chromosomal analyses, they were fixed in Carnoy’s solution (methanol and acetic acid at a ratio of 3:1) and were subsequently stored untouched in freezers (−20ºC) for more than 10 years. The chromosomal suspensions were prepared from kidney cells in 2010, following the protocols described by Bertollo et al. [9] and Foresti et al. [10]. The samples were then stored in the collections of the Research Group on the Fish of the Middle Araguaia Region (GEPEMA) and the Laboratory of Cytogenetics and Animal Genetics (LABGEN) at the Federal University of Mato Grosso in Cuiabá, Brazil.

### Extraction of the DNA from cells fixed in Carnoy’s solution

The protocol described in this peer-reviewed article is published on protocols.io (https://dx.doi.org/10.17504/protocols.io.e6nvw4289lmk/v1) and is included for printing purposes as S1 File.

Cell suspensions prepared for cytogenetic analyses and stored at −20°C were vortexed for 10 seconds. A 150 μL aliquot of each sample was pipetted into a new, pre-labeled 1.5 mL microcentrifuge tube and centrifuged at 8,000 rpm for 10 minutes. After the supernatant was discarded, the tube was placed in an incubator at 37°C for 45 minutes, to ensure the complete evaporation of the fixative.

Subsequent DNA extraction steps followed the protocol of Aljanabi and Martinez [1] with the modifications detailed below. To begin with, 440 μL of extraction buffer was added to each tube. This buffer contained 8 mL of 5 M NaCl, 1 mL of 1 M Tris-HCl (pH 8.0), 400 μL of 0.5 M EDTA (pH 8.0), 20 mL of 10% SDS, and ultrapure water for a final volume of 100 mL. A further 16 μL of proteinase K (10 mg.mL^-1^) was also added to each tube at this stage. The contents of the tubes were then vortexed to ensure homogenization and incubated in a dry bath at 55°C for 1 hour and 30 minutes.

Subsequently, 300 μL of 5 M NaCl were added to each tube, followed by inversion and vortexing for 30 seconds. The samples were then centrifuged at 10,000 rpm for 10 minutes. An aliquot of 500 μL of the supernatant of each sample was then transferred to a new 1.5 mL microtube and mixed with 500 μL of ice-cold 100% isopropanol. The tubes were then inverted gently to precipitate the DNA, and centrifuged once again at 10,000 rpm for 10 minutes. The supernatant was discarded, and the resulting pellet was washed with 300 μL of ice-cold 70% ethanol, and then centrifuged 10,000 rpm for 5 minutes. The ethanol was discarded, and the DNA pellet was air-dried in an incubator at 37°C for one hour. In the final step, the DNA was resuspended in 30 μL of autoclaved ultrapure water.

Following extraction, the concentration and purity of the DNA were assessed using a spectrophotometer (DENOVIX), with the results being expressed in nanograms per microliter (ng.μL^-1^). The integrity of the DNA was then evaluated by electrophoresis in 1.5% agarose gel, using a horizontal electrophoresis system with 1 × TBE buffer. A 1 Kb molecular ladder (Kasvi) was included for reference. For the analysis of each sample, 2 μL of the extracted DNA were combined with 1 μL of GelRed™ fluorescent dye (20 × , Biotium) and 1 μL of Blue Juice loading dye. The samples were then electrophoresed at 120 volts and 240 milliamps for 30 minutes, and the DNA bands were visualized under an ultraviolet light using a Loccus Biotechnology photo-documentation system.

### Evaluation of the Efficiency of the DNA Extraction Protocol by Polymerase Chain Reaction (PCR)

The efficiency of the extraction of the DNA obtained from the cytogenetic cell suspensions was assessed by amplifying four different genes by Polymerase Chain Reaction (PCR). These genes were the mitochondrial cytochrome oxidase subunit I (COI) and three nuclear markers, 5S rDNA, 18S rDNA, and RAG2 (Table 1). The PCRs were run in a final volume of 25 μL, containing: 17.9 μL of ultrapure water, 2.5 μL of 10 × buffer, 1.5 μL of dNTP mix (1.25 mM), 1 μL of MgCl₂ (50 mM), 0.5 μL of each primer, 0.1 μL of Taq DNA polymerase (Platinum®, Invitrogen; 5 U/μL), and 1 μL of the DNA template extracted from each sample. The samples were amplified in a Veriti® thermal cycler (Applied Biosystems) with the following cycling conditions: initial denaturation at 95°C for 3 minutes, followed by 35 cycles of denaturation at 95°C for 30 seconds, annealing at 50–55°C for 45 seconds, and extension at 72°C for 50 seconds, with a final extension at 72°C for 7 minutes.

**Table 1:** The genes and primer sequences used for the amplification of the samples analyzed in the present study, in the 5’ → 3’ direction.

Gene	Primer	Sequence	Reference
**COI**	FISH F1	TCAACCAACCACAAAGACATTGGCAC	[11]
	FISH R1	TAGACTTCTGGGTGGCCAAAGAATCA	[11]
**RAG2** **1st PCR**	164F	AGCTCAAGCTGCGYGCCAT	[12]
	RAG2-R6	TGRTCCARGCAGAAGTACTTG	[13]
**RAG2** **2nd PCR**	176R	GYGCCATCTCATTCTCCAACA	[12]
	RAG2 Ri	AGAACAAAAGATCATTGCTGGTCGGG	[12]
**5S**	5S-A	TACGCCCGATCTCGTCCGATC	[14]
	5S-B	CAGGCTGGTATGGCCGTAAGC	[14]
**18S**	NS1	GTAGTCATATGCTTGTCTC	[15]
	NS8	TCCGCAGGTTCACCTACGGA	[15]

The PCR products were visualized by electrophoresis in 1.5% agarose gel, using the same protocol as that used for the assessment of the integrity of the DNA, except for the inclusion of a 100-bp molecular ladder, rather than a 1Kb reference marker. The samples were electrophoresed at for 40 minutes at 90 volts and 240 milliamps.

The mitochondrial cytochrome c oxidase subunit I (COI) gene was amplified from all 27 of the DNA samples extracted from the cytogenetic suspensions analyzed here. In the case of the nuclear markers, a test was done with a few samples just to check the efficiency of DNA extraction, for the ribosomal genes (5S and 18S rDNA) were each amplified from only two samples, while the Recombination-Activating Gene 2 (RAG2) was amplified from a single sample.

## Results and discussion

**Fig 1:**
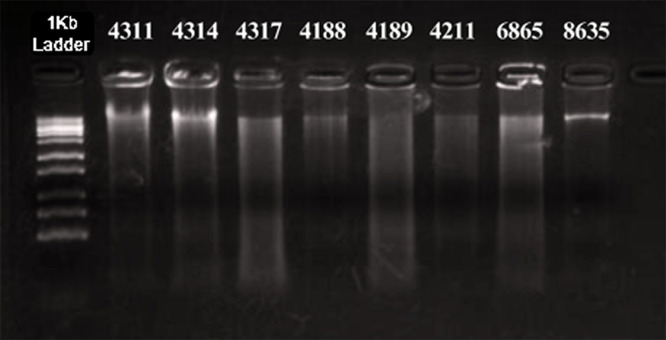
Electrophoretic gel showing the quality of the genomic DNA extracted from the samples (cytogenetic suspensions) processed in the present study. The sample numbers correspond to those in Table 2. **Electrophoresis was performed under constant voltage (120 V) for 30 minutes, using a 1 Kb DNA ladder as molecular size marker.**

The technique that was adapted here for the extraction of DNA from the cytogenetic preparations fixed in Carnoy’s solution yielded satisfactory results, with the extracted DNA being of good quality and available at high concentrations (Fig 1). The spectrophotometric readings indicated DNA concentrations ranging from 5.902 ng.µL^-1^ to 1739.05 ng.µL^-1^, with most samples in the range of less than 100 ng.µL^-1^ range (Table 2). The purity of the extracted DNA was confirmed by absorbance ratios (A260/A280) of 1.75–2.07 across all samples, indicating low protein contamination. Absorbance ratios of between 1.7 and 2.0 are generally considered to be adequate for analytical applications in molecular biology [16]. Regitano [17] also reported that ratios as low as 1.4 can be considered satisfactory for the extraction of DNA from fresh blood using the salting-out method described by Olerup & Zetterquist [18], which provides samples adequate for PCR amplification.

**Table 2:** Molecular data and BOLD Systems accession numbers for the analyzed specimens. Specimens marked with an * were not sequenced by Sanger.

Species	Sample identification code	DNA concentration	A260/A280	Amplified genes	Bold Systems access number (COI)
*Rineloricaria parva*	LABGEN-8313	32.114	1.75	COI	SUBLO026−25
*Rineloricaria parva*	LABGEN-8315	97.353	1.89	COI	SUBLO027−25
*Rineloricaria parva*	LABGEN-8316	13.54	1.74	COI	SUBLO028−25
*Rineloricaria parva*	LABGEN-8333	31.283	1.99	COI	SUBLO029−25
*Rineloricaria lanceolata*	LABGEN-8635	24.553	2.07	COI	SUBLO050−25
*Rineloricaria lanceolata*	LABGEN-8636	47.309	1.72	COI	SUBLO051−25
*Loricaria* cf. *lata*	GEPEMA-4188	17.579	1.71	COI	SUBLO044−25
*Loricaria* cf. *lata*	GEPEMA-4189	210.515	1.82	COI	SUBLO045−25
*Loricaria* cf. *lata*	GEPEMA-4191	6.904	1.85	COI	SUBLO053−25
*Loricaria* sp. 1	GEPEMA-4192	84.359	1.79	COI	SUBLO018–25
*Loricaria* sp. 1	GEPEMA-4193	15.726	1.96	COI	SUBLO019–25
*Loricaria* sp. 2	GEPEMA-4211	11.139	2.01	COI	SUBLO020–25
*Sturisoma ghazziae*	GEPEMA-4213	179.022	1.97	COI, RAG2	*
*Spatuloricaria* sp. 1	GEPEMA-4220	66.859	2.07	COI	SUBLO034−25
*Loricariichthys* cf. *nudirostris*	GEPEMA-4224	273.283	1.83	COI	SUBLO046−25
*Spatuloricaria* sp. 1	GEPEMA-4310	1375.344	1.92	COI, 5S, 18S	SUBLO035−25
*Spatuloricaria* sp. 1	GEPEMA-4311	770.557	1.79	COI	SUBLO036−25
*Loricaria* sp. 3	GEPEMA-4314	419.127	1.81	COI	SUBLO021–25
*Spatuloricaria* sp. 1	GEPEMA-4315	1739.05	1.91	COI	SUBLO037−25
*Spatuloricaria* sp. 1	GEPEMA-4316	18.777	1.51	COI	*
*Sturisoma* sp. 1	GEPEMA-4317	686.664	1.78	COI	SUBLO052−25
*Spatuloricaria* sp. 1	GEPEMA-4318	97.248	1.87	COI	SUBLO038−25
*Loricaria* sp. 3	GEPEMA-6513	17.432	1.91	COI	SUBLO022–25
*Rineloricaria* sp. 2	GEPEMA-6864	5.902	1.82	COI	SUBLO032−25
*Spatuloricaria* sp.	GEPEMA-6865	58.686	1.86	COI, 5S, 18S	SUBLO032−25
*Spatuloricaria* sp. 2	GEPEMA-6866	38.789	1.86	COI	SUBLO033−25
*Loricariichthys* cf. *nudirostris*	GEPEMA-6868	75.089	1.8	COI	SUBLO023–25

The collection of high-quality genomic DNA is a fundamental and critical step in molecular biology, which can have a significant effect on the outcome of any subsequent analyses [2]. In particular, the extracted material should contain negligible amounts of proteins, RNA and any other substance that might inhibit the PCR, and the fragmentation of the DNA during the extraction process should be avoided as far as possible [19]. The quality and integrity of the DNA obtained here (Fig 1) indicate that these objectives were achieved effectively during the extraction process tested in the present study.

Appropriate procedures for the storage of the tissue are essential to prevent possible degradation and guarantee the extraction of stable DNA of good quality at high concentrations. Ideally, tissue samples should be preserved in absolute ethanol and stored at temperatures of between –20°C and –80°C to ensure the integrity of the DNA [20]. When material of this type is lacking, samples prepared for other purposes – such as the cytogenetic suspensions analyzed here – can possibly be used, depending on the availability of alternative methods for the extraction of the DNA. The results of the present study have shown that Carnoy’s solution, which contains methanol and acetic acid in the ratio of 3:1, preserves chromosomal structure without compromising the integrity of the DNA significantly. Cytogenetic suspensions fixed in Carnoy’s solution and stored at –20°C for over 10 years were successfully processed, demonstrating that this procedure can yield high-quality DNA for molecular analyses from alternative sources.

**Fig 2:**
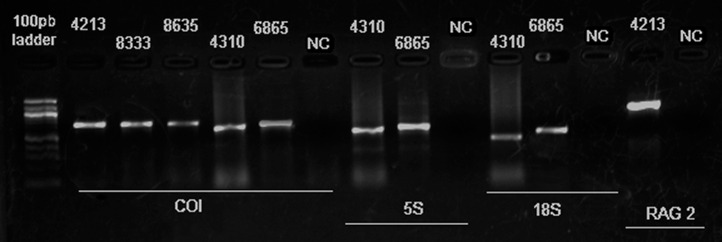
Electrophoretic gel showing the PCR products generated by the primers for the COI, 5S rDNA, 18S rDNA, and RAG2 genes, applied to DNA extracted from samples of cytogenetic suspension fixed in Carnoy’s solution. The sample numbers correspond to those in Table 2. **NC = Negative Control. Electrophoresis was performed at 90 V for 40 minutes, using a 100 bp DNA ladder as molecular size marker.**

In order to assess more fully the integrity of the genomic DNA obtained using the new method, four genes targeted frequently in laboratory analyses – COI, 5S rDNA, 18S rDNA, and RAG2 – were amplified from the samples by PCR (Table 2). The PCRs were successful for all the primers tested (Fig 2), which indicates a lack of inhibitory substances in the extracted DNA. This confirms the viability and high quality of the DNA for use in molecular studies.

The PCR technique amplifies specific segments of the DNA, and supports a wide range of molecular studies, including the development of highly sensitive diagnostic methods, the production of DNA for sequencing, and the analysis of the genetic diversity in populations [20]. While the PCR is a relatively simple procedure, it depends on the adequate adjustment of DNA extraction protocols to ensure the availability of good quality DNA. In particular, the appropriate adjustment of the extraction parameters to obtain good-quality DNA from material processed originally for other purposes will be essential to ensure the availability of DNA of adequate quality for successful PCR amplifications [21].

Once amplified, the DNA extracted using the method described here was sequenced by the Sanger method. In the specific case of the COI gene, a 623 bp fragment was recovered, with quality scores of over 90% for all 27 samples, the sequences were previously deposited in BOLD (Table 2). This further highlights the effectiveness of the proposed DNA extraction method, and its suitability for a number of different areas of molecular biology.

Fixatives or certain types of preservatives can compromise the quality of the nucleic acids extracted from a sample, given the potential for chemical interactions [22]. However, the evidence presented here indicates that the use of Carnoy’s solution did not degrade the samples, which contained amplifiable DNA even after long-term storage.

## Conclusions

The present study describes an alternative protocol for the extraction of samples of genomic DNA from stored cytogenetic suspensions fixed in Carnoy’s solution (three parts methanol to one part acetic acid). The protocol proved to be highly efficient, yielding extremely pure DNA at a satisfactory concentration, with results comparable with conventional methods used to extract DNA from muscle tissue. The proposed protocol therefore represents a viable alternative for obtaining high-quality DNA when fresh samples are scarce or difficult to collect, while also reducing cell lysis time. This makes the extraction process faster, more practical, and more cost-effective.

## Supporting information

S1 File Step-by-step protocol, also available on protocols.io. (PDF)

S1 images raw Unedited images corresponding to Figs 1 and 2. (PDF)
