# Supplementary material for: Efficient DNA extraction from cytogenetic suspensions: A new possibility for obtaining DNA, with potential applications in studies of molecular markers
Source: PLoS One. 2025 Nov 7;20(11):e0335898. doi: 10.1371/journal.pone.0335898 (PMC12594425; doi:10.1371/journal.pone.0335898)
Supplement: S1 File — (PDF) [file pone.0335898.s001.pdf]

Oct 10, 2025 Version 1

Efficient DNA extraction from cytogenetic suspensions: a new possibility for obtaining DNA, with potential applications in studies of molecular markers V.1

DOI
dx.doi.org/10.17504/protocols.io.e6nvw4289lmk/v1

Geórgia Liz Monteiro Sant'ana-Arruda[1], Paulo Cesar Venere[2], Daniela Cristina Ferreira[2]

[1]Graduate Program in Ecology and Conservation of Biodiversity, Universidade Federal de Mato Grosso, campus Universitário de Cuiabá, Avenida Fernando Correia da Costa, 2367, Cuiabá, Mato Grosso, Brazil.;
[2]Department of Biology and Zoology, Universidade Federal de Mato Grosso, campus Universitário de Cuiabá, Avenida Fernando Correia da Costa, 2367, Cuiabá, Mato Grosso, Brazil.

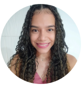

Geórgia Liz Monteiro Sant'ana-Arruda
Universidade Federal de Mato Grosso

sdsdsCreate & collaborate more with a free account

Edit and publish protocols, collaborate in communities, share insights through comments, and track progress with run records.

Create free account

OPEN ACCESS

DOI: https://dx.doi.org/10.17504/protocols.io.e6nvw4289lmk/v1

Protocol Citation: Geórgia Liz Monteiro Sant'ana-Arruda, Paulo Cesar Venere, Daniela Cristina Ferreira 2025. Efficient DNA extraction from cytogenetic suspensions: a new possibility for obtaining DNA, with potential applications in studies of molecular markers. **protocols.io https://dx.doi.org/10.17504/protocols.io.e6nvw4289lmk/v1**

License: This is an open access protocol distributed under the terms of the **Creative Commons Attribution License**, which permits unrestricted use, distribution, and reproduction in any medium, provided the original author and source are credited

Protocol status: Working
We use this protocol and it's working

Created: October 10, 2025

Last Modified: October 10, 2025

Protocol Integer ID: 229514

Keywords: dna extraction method from cytogenetic suspension, efficient dna extraction from cytogenetic suspension, dna extraction, extracted dna, dna extraction method, efficient dna extraction, cytogenetic suspension, obtaining dna, extraction, suitable for downstream molecular analysis, molecular marker, molecular markers the protocol, downstream molecular analysis, dna, involving enzymatic digestion, enzymatic digestion, ethanol washing

Funders Acknowledgements:
Instituto Nacional de Ciência e Tecnologia (INCT-Peixes), funded by MCTIC/CNPq
Grant ID: 405706/2022-7
Conselho Nacional de Desenvolvimento Científico e Tecnológico-CNPq
Grant ID: 421733/2017-9
Fundação de Amparo a Pesquisa do Estado de Mato Grosso
Grant ID: PRO.000339/2023

Disclaimer

DISCLAIMER – FOR INFORMATIONAL PURPOSES ONLY; USE AT YOUR OWN RISK
The protocol content here is for informational purposes only and does not constitute legal, medical, clinical, or safety advice, or otherwise; content added to **protocols.io** is not peer reviewed and may not have undergone a formal approval of any kind. Information presented in this protocol should not substitute for independent professional judgment, advice, diagnosis, or treatment. Any action you take or refrain from taking using or relying upon the information presented here is strictly at your own risk. You agree that neither the Company nor any of the authors, contributors, administrators, or anyone else associated with **protocols.io**, can be held responsible for your use of the information contained in or linked to this protocol or any of our Sites/Apps and Services.

Abstract

The protocol describes a DNA extraction method from cytogenetic suspensions fixed in Carnoy's solution (methanol and acetic acid at a ratio of 3:1) and stored at -20°C for more than 10 years, involving enzymatic digestion, isopropanol precipitation, and ethanol washing. After the final resuspension, the extracted DNA is expected to show good integrity and purity, suitable for downstream molecular analyses such as PCR or sequencing.

Materials

Reagents and Solutions

- Carnoy's fixative solution (3:1 methanol:glacial acetic acid);
- Extraction buffer (prepare 100 mL with):
  NaCl 5 M – 8 mL
  Tris-HCl 1 M pH 8.0 – 1 mL
  EDTA 0.5 M pH 8.0 – 400 µL
  SDS 10% – 20 mL
  Ultrapure (Milli-Q) water – complete to 100 mL (≈70.6 mL);
- Proteinase K (10 mg/mL);
- NaCl 5 M (stock solution);
- 100% isopropanol (ice-cold);
- 70% ethanol (ice-cold);
- Autoclaved Milli-Q water;

Laboratory Materials

- 1.5 mL microcentrifuge tubes (sterile and labeled);
- Micropipette tips (10 µL, 200 µL, 1000 µL; sterile);
- Micropipettes (10 µL, 200 µL, 1000 µL);
- Vortex mixer;
- Bench centrifuge (capable of reaching 10,000 rpm);
- Water bath (set to 55 °C);
- Incubator (set to 37 °C);
- Tube rack;
- Absorbent paper (for drying tubes/pellets);
- Personal protective equipment (PPE): lab coat, gloves, safety goggles
- Spectrophotometer or NanoDrop (to check DNA purity and concentration)
- Agarose gels and electrophoresis equipment (to assess DNA integrity)

Troubleshooting

1 Homogenize the cytogenetic suspensions fixed in Carnoy's solution (methanol and acetic acid at a ratio of 3:1) .

2 Transfer 150 µL to a 1.5 mL microcentrifuge tube.

3 Centrifuge at 8,000 rpm for 10 minutes.

4 Discard the supernatant and allow the fixative to evaporate in an incubator at 37 °C for 45 minutes (or until no odor remains).

5 Add 440 µL of extraction buffer (NaCl 5 M: 8 mL; Tris-HCl 1 M pH 8.0: 1 mL; EDTA 0.5 M pH 8.0: 400 µL; SDS 10%: 20 mL; ultrapure water: 70.6 mL; final volume: 100 mL) and 16 µL of proteinase K (10 mg/mL).

6 Vortex briefly and incubate in a water bath at 55 °C for 1 hour 30 min.

7 Add 300 µL of NaCl 5 M. Vortex for 30 seconds.

8 Centrifuge at 10,000 rpm for 10 minutes. Transfer 500 µL of the supernatant to a previously labeled 1.5 mL microcentrifuge tube.

9 Add 500 µL of ice-cold 100% isopropanol. Mix by gentle inversion.

10 Centrifuge at 10,000 rpm for 10 minutes. Discard the supernatant.

11 Wash the pellet with 300 µL of ice-cold 70% ethanol.

12 Centrifuge at 10,000 rpm for 5 minutes. Discard the supernatant and allow the pellet to air-dry.

13 Resuspend the pellet in 30 µL of autoclaved Milli-Q water.

Protocol references

Aljanabi SM, Martinez I. Universal and rapid salt-extraction of high quality genomic DNA for PCR-based techniques. Nucleic Acids Res. 1997;25: 4692–4693.
