# Supplementary figures and images for: Efficient DNA extraction from cytogenetic suspensions: A new possibility for obtaining DNA, with potential applications in studies of molecular markers
Source: PLoS One. 2025 Nov 7;20(11):e0335898. doi: 10.1371/journal.pone.0335898 (PMC12594425; doi:10.1371/journal.pone.0335898)

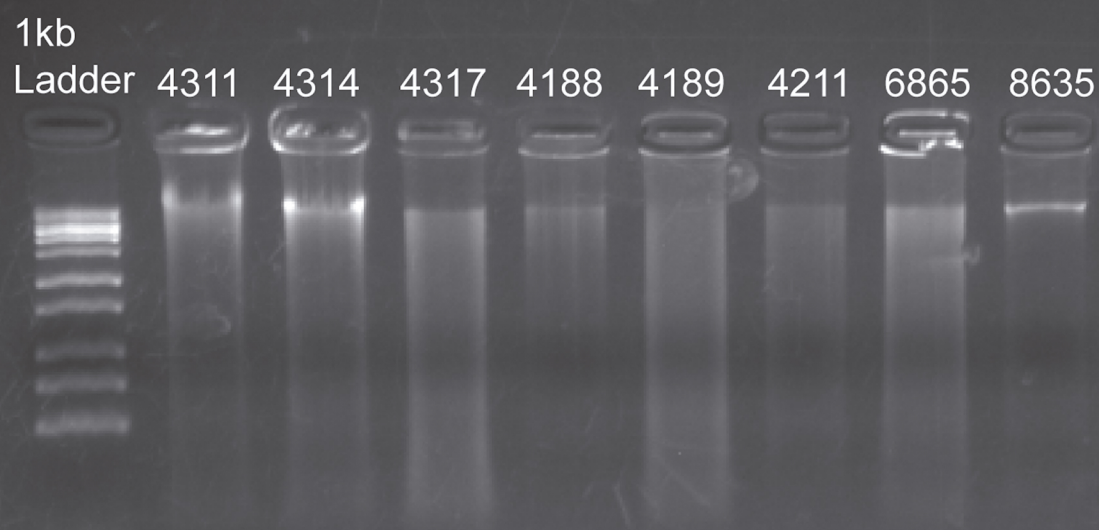

Figure 1

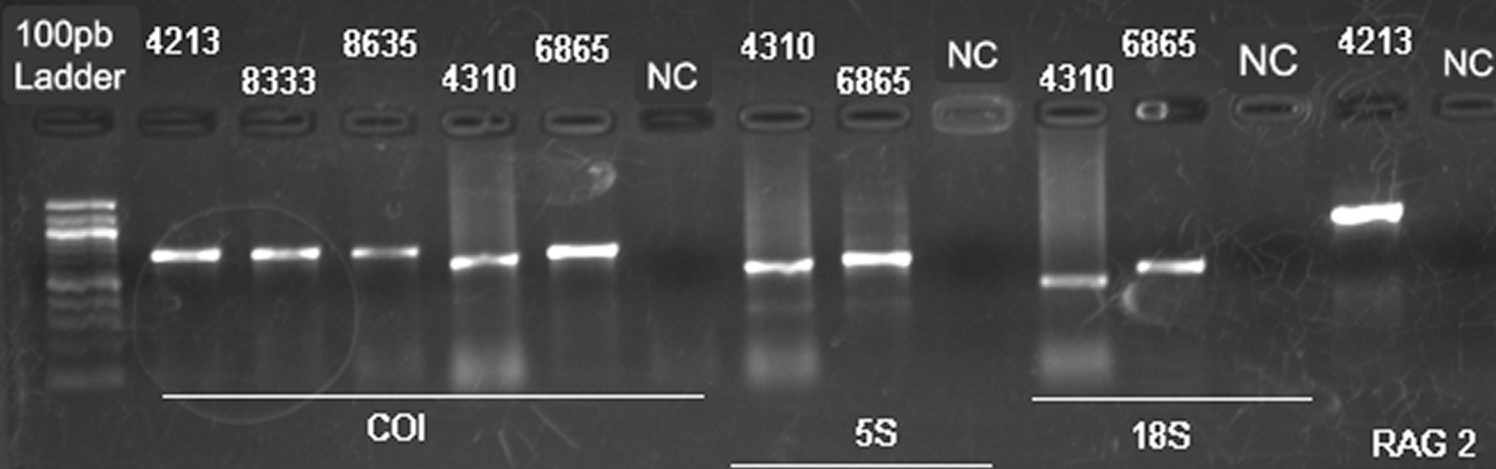

Figure 2

Supplement: S1 images raw — (PDF) [file pone.0335898.s002.pdf]
